# M. Deleau on the Eustachian Tube

**Published:** 1831-04-01

**Authors:** 


### On the Employment of Atmospheric Air as a Mean of Diagnosis, Prognosis, and Treatment of Deafness occasioned by Chronic Diseases of the Eustachian Tube. By M. Deleau, Junr.

In a late number of Magendie's journal, there is an extended memoir on the above subject, said to be founded on much practical observation and experience.

The author observes that every time we inspire there is some introduction of air through the Eustachian tube into the cavity of the tympanum. This, he thinks, may be proved by the sensations of any individual who inspires strongly on going from a warm to a cold air. He will then feel the cold in the interior of the ear. In the act of sneezing and blowing the nose, the access of air to the cavity of the tympanum is facilitated. A person burst the membrana tympani by violent straining at stool. When a person is partially deaf from cold, the hearing is bettered by blowing the nose. These and various other considerations, M. Deleau thinks, authorise us to conclude that the delicacy of hearing depends in a great measure on the free circulation of air in the cavity of the tympanum through the Eustachian tube—and consequently that, when this passage is obstructed by any means, the want of fresh air in the said cavity, becomes a source of deafness.

M. Deleau alludes to the fact that hearing is not affected by perforation of the membrana tympani, until inflammation arises, which it always does af-

---

ter lesion of this auditory diaphragm. He conceives that the motions or vibrations of the membrana tympani contribute to move the air in the cavity of the ear, and thus secure its renovation. So the presence of wax in the meatus auditorius causes deafness, not only by intercepting the sound, but by depriving the membrana tympani of its elasticity and motions, and thus preventing the renovation of air in the internal ear. Polypi and chronic inflammation of the auditory passages produce the same effect. Among the causes of deafness may be remarked tumefactions of the tonsils, which obstruct the Eustachian tubes, and prevent the access of air to the internal ear. This effect is particularly observable in children, after measles, scarlatina, and inflammations of the air-passages. The same effect is also observable in people of all ages, after or during diseases about the throat which obstruct the Eustachian tubes.

The author then proceeds to remark that it is only in chronic affections of the ear, that we should think of injecting warm air into the Eustachian tubes. This operation is to the ear, he observes, what catheterism is to the urethra and bladder. Inflammation, he thinks, cannot subsist long in the Eustachian tube, without producing more or less of stricture in that canal. M. Deleau avers that he is able to recognise the physiological and pathological condition of the Eustachian tube and cavity of the tympanum, by means of air thrown in through a gum-elastic tube —and that by the nature of the sounds which are produced by this introduction —by the effects resulting in respect to audition—and the sensibility of the parts to air.

He informs us that, if air be thrown into the Eustachian tube of a person in health, the individual immediately claps his hand on the external ear, and feels as if water were injected into the meatus auditorius, with all the strange and tumultuous noises thence resulting, occasioning some alarm in his mind. M. Deleau says, that if the ear of the operator be applied to that of the person operated on, the noise appears to be reverberated on his own tympanum, and resembles the distant sound of a cascade, or a fall of rain in a wood. He goes on to describe the various sounds and phenomena resulting from this operation, and the conclusions which may be drawn from them; but we are unable to follow him. Those who practise exclusively on the ear will probably do well to consult this essay of M. Deleau, in the journal which we have indicated.

